# Experiments on Living Animals

**Published:** 1909-06-26

**Authors:** 


### The Hospital

A Journal of

The Medical Sciences and Hospital Administration.

New Series. No. 122, Vol. V. [No. 1194, Vol. XLVI.] SATURDAY, JUNE 26, 1909.

### Experiments on Living Animals.

On Monday June 21 the Home Office issued a return, showing the number, range and character of the experiments performed upon living animals during 1908, by licensees under the Act of 1876. This return distinguishes the nature of the various experiments performed during the year, and it is issued as a Parliamentary Paper. The appearance of this report on the longest day of the year is not altogether inappropriate. Public attention has lately been drawn, by a number of different forces, towards the whole subject of experimental research, which certainly deserves careful consideration from every responsible citizen. The conflicting claims of the various rival anti-vivisection societies, and of the newly formed but already powerful organisation, called the Research Defence Society, have already brought the matter into the category of questions of the day.

The public seems already to have grasped the great essential principle which underlies the subject of vivisection: namely that facts, and facts only, are needed for the formation of a settled opinion. The Home Office return bristles with facts, and is therefore an invaluable asset to the somewhat uphill activities of the Research Defence Society, whose annual general meeting, under the chairmanship of its distinguished president, Lord Cromer, is taking place at the house of the Royal Society of Medicine at the time this number appears. The inspector's report for England and Scotland naturally occupies the greater share of the return, since the great majority of experiments upon living animals in these islands are conducted upon this side of St. George's Channel. This however must not be interpreted as a sign that educated Irish opinion in any degree dissociates itself from the view that experiments upon animals are vitally necessary for the maintenance of medical and scientific progress. The inaugural meeting, early in the present year, of the Dublin branch of the Research Defence Society, showed clearly enough that in this matter Ireland is at one with the larger island.

The purpose which we have in view, and which should by now be well known to our readers, will be served best by the publication here of a few short extracts from the Home Office return. These will give the defenders of the honour and potential strength of the medical profession material for personal satisfaction, and—what is at the moment of far greater importance—material wherewith to combat and defeat the insidious attacks of the enemies of truth, progress and true humanity.

The report for England and Scotland shows that all licensees were restricted to the registered place or places specified on their licenses, with the exception of those who were permitted to perform inoculation experiments in situations other than "registered places," with the object of studying outbreaks of disease occurring in remote districts or in circumstances rendering it impracticable to perform the experiment in a "registered place." The total number of licensees was 453, and of these 126 performed no experiments. As is prominently pointed out in *The Times* of June 22, it is perfectly clear, from the tabulated returns of registered places and licensees, that licenses and certificates have been granted only upon the recommendation of persons of high scientific standing; that the licensees are persons who, by their training and education, are fitted to undertake experimental work and to profit by it; and that all experimental work has been conducted in suitable places, and under proper conditions. The total number of experiments was 88,634, being 15,260 more than in 1907; but this increase is largely due to the greater number of simple inoculations, which were more numerous than in 1907 by 14,989. The balance of the increase was accounted for by 271 experiments, all performed under anæsthesia. Every experiment involving a serious operation, 1,615 in all, came under the provision of the Act that the animal must be kept under an anæsthetic during the whole of the experiment, and must, if the pain is likely to continue after the cessation of anæsthesia, or if serious injury has been inflicted, be killed before it recovers. In the experiments performed under special certificates, 1,236 in number, the initial operations are performed under anæsthetics, from the influence of which the animals are allowed to recover. The operations are required to be performed antiseptically, the report states, so that the healing of the wounds shall, as far as possible, take place without pain. If the antiseptic precautions fail, and suppuration occurs, the animal is required to be killed.

Eighty-five thousand seven hundred and eighty-three experiments were performed without anæsthetics; but these were mostly inoculations; and in no instance was a certificate dispensing with anæsthesia allowed for an experiment involving a serious operation.

In the significant words of the return, the irregularities which occurred during the year were few, and all arose from misunderstanding or inadvertence with respect to the extent or application of certificates. Finally, with regard to Ireland, the inspector reports that the work performed there in 1908 under the Act was, in his opinion, free from abuse, and was sincere and well-intentioned. Thus may be dispelled, by the light of cold, quiet facts, the shadowy horrors circulated by unprincipled agitators.

